# Supplementary material for: Higher operating theatre temperature during burn surgery increases physiological heat strain, subjective workload, and fatigue of surgical staff
Source: PLoS One. 2023 Jun 2;18(6):e0286746. doi: 10.1371/journal.pone.0286746 (PMC10237492; doi:10.1371/journal.pone.0286746)
Supplement: S1 Table — (PDF) [file pone.0286746.s001.pdf]

	Males	Females	Surgeons	Scrub nurses	Registrars	Age (years)	Height (cm)	Mass (kg)
**CON**	2	5	4	3		50 ± 8	165.3 ± 5.8	79.5 ± 17.0
**HOT**	3	7	5	3	2	45 ± 10	166.6 ± 5.5	79.9 ± 16.4
