# Supplementary material for: Higher operating theatre temperature during burn surgery increases physiological heat strain, subjective workload, and fatigue of surgical staff
Source: PLoS One. 2023 Jun 2;18(6):e0286746. doi: 10.1371/journal.pone.0286746 (PMC10237492; doi:10.1371/journal.pone.0286746)
Supplement: S3 Table — All data expressed as Mean ± SD. (PDF) [file pone.0286746.s003.pdf]

	Dominant Hand		Non-Dominant Hand	
	PRE	*POST*	*PRE*	*POST*
**CON**	18 ± 1	19 ± 1	17 ± 1	17 ± 2
**HOT**	18 ± 2	19 ± 2	17 ± 2	17 ± 1
