# Supplementary material for: Higher operating theatre temperature during burn surgery increases physiological heat strain, subjective workload, and fatigue of surgical staff
Source: PLoS One. 2023 Jun 2;18(6):e0286746. doi: 10.1371/journal.pone.0286746 (PMC10237492; doi:10.1371/journal.pone.0286746)
Supplement: S4 Table — (PDF) [file pone.0286746.s004.pdf]

	PRE				POST			
	Well hydrated	Minimal dehydration	Significant dehydration	Serious dehydration	Well hydrated	Minimal dehydration	Significant dehydration	Serious dehydration
Control	**12** *(55)*	**6** *(27)*	**4** *(18)*	**0** *(0)*	**2** *(10)*	**12** *(57)*	**7** *(33)*	**0** *(0)*
Hot	**8** *(50)*	**7** *(44)*	**1** *(6)*	**0** *(0)*	**0** *(0)*	**6** *(43)*	**7** *(50)*	**1** *(7)*

[1] It is important to note that measurement of USG may not reflect plasma osmolality, the most efficient measure to assess hydration status, and so the classifications provided may not be accurate in illustrating the extent of hypohydration

[1] Pereira ER, de Andrade MT, Mendes TT, Ramos GP, Maia-Lima A, Melo ES, et al. Evaluation of hydration status by urine, body mass variation and plasma parameters during an official half-marathon. J Sports Med Phys Fitness. 2017; 57(11): 1499-1503. Doi: 10.23736/s0022-4707.16.06836-5
