# Supplementary material for: Higher operating theatre temperature during burn surgery increases physiological heat strain, subjective workload, and fatigue of surgical staff
Source: PLoS One. 2023 Jun 2;18(6):e0286746. doi: 10.1371/journal.pone.0286746 (PMC10237492; doi:10.1371/journal.pone.0286746)
Supplement: Attachment — Submitted filename: Response to reviewers 22.05.23.docx [file pone.0286746.s014.docx]

**GENERAL COMMENTS**

The authors have addressed all the comments made by the reviewers satisfactorily. Indeed, the authors were highly responsive to these comments. Thank you! As a result, the revised manuscript was much improved compared to its first version. However, a few minor modifications should be made before the study is ready for publication; please see the Academic Editor’s comments at the end of this letter.

Reviewer #1: I was very pleased with the authors' review of the article and their responses to my questions. Congratulations on the nice article!

Reviewer #3: The authors addressed my comments satisfactorily. Congratulations!
Thank you for the opportunity to review this manuscript.
Kind Regards.

**Response: We thank the reviewers for their positive feedback on the manuscript.**

**JOURNAL REQUIREMENTS:**

Please review your reference list to ensure that it is complete and correct. If you have cited papers that have been retracted, please include the rationale for doing so in the manuscript text, or remove these references and replace them with relevant current references. Any changes to the reference list should be mentioned in the rebuttal letter that accompanies your revised manuscript. If you need to cite a retracted article, indicate the article’s retracted status in the References list and also include a citation and full reference for the retraction notice.

Response:

The reference list has been reviewed and is complete and correct. No retracted papers have been cited in the mnuscript.

**MINOR REVISIONS**

**Abstract, line 50. Please consider referring to the HOT condition as approximately 31°C heat, not 30°C.**

Response:

Lines 49-51 of the abstract now read: “*Cognitive function and manual dexterity were mainained in hot conditions, suggesting that operating in approximately 31°C heat is a safe approach for patient treatment.*

**Methods, line 145. Please consider replacing “TCORE and HR measurement was taken as soon” with “TCORE and HR measurements were taken as soon”.**

Response:

Line 145 of the methods section now reads: *“T_CORE_ and HR measurements were taken as soon as surgery commenced...”*

**Please describe the results in a better way. For example, in the paragraph between lines 211 and 217, the authors could merge the two sentences about interaction into one sentence. Suggestion: “There was no interaction between theatre temperature and time on counting latency (p = .203; Fig 1) or time on the number of correct responses (p = .757; Table 1)”.**

Response:

Lines 215-217 of the results section now read: *“There was no interaction between theatre temperature and time on counting latency (p = .203; Fig 1) or the number of correct responses (p = .757; Table 1)”.*

**Similarly, in the paragraph between lines 230 and 239, the authors could merge the three sentences about interaction into only one sentence. Suggestion: “There was no interaction between theatre temperature and time on recall latency (p = .821; Fig 2), number of correct responses (p = .828; Table 1), or overall counting span score (p = .949; Table 1).**

Response:

Lines 235-237 of the results section now read: *“There was no interaction between theatre temperature and time on recall latency (p = .821; Fig 2), number of correct responses (p = .828; Table 1), or overall counting span score (p = .949; Table 1).*

**Results, lines 262 to 266. It is challenging to understand the information in the following sentence: “There was an interaction between theatre temperature and time on TS (p = .019), TC (p = .047), and RPE (p < .001), which indicated similar perceptual responses for TS and RPE in CON and HOT pre-surgery (p > .924), but a higher TC score pre-surgery in HOT (11 ± 3) than CON (8 ± 4; p = .025).” Please amend this sentence to improve clarity. The authors may want to focus on post-surgery rather than on pre-surgery data when describing the findings concerning these three perceptual variables. In the case of TC, it is also valid to comment that a difference already exists before surgery.**

Response:

Thank you for your comment. To clarify, the post-surgery perceptual responses have been explained in detail in respect to the main effect for time. A sentence has been added to the discussion of the interaction to explain the post-surgery comparison between CON and HOT. The statement regarding the interaction discusses pre-surgery as that is where the significant post-hocs were found. To improve clarity, lines 260-264 of the manuscript now read: *“There was an interaction between theatre temperature and time on TS (p = .019), TC (p = .047), and RPE (p < .001). The interaction supported that scores post-surgery were higher in HOT than CON for all perceptual measures (p < .001). It also revealed that ratings of TS and RPE were the same pre-surgery in CON and HOT (p > .924), but a difference in ratings of TC existed before surgery (HOT: 11 ± 3, CON: 8 ± 4; p = .025)”.*

**Please indicate where the Discussion section starts. The revised manuscript lacks the heading of the discussion section.**

Response:

The heading “*Discussion*” has now been added to line 314 of the manuscript, to indicate where the discussion section starts.

**The figures are not shown in an appropriate order in the pdf file of the revised manuscript. For example, figure 6 comes before figures 1 and 2. Please amend this issue to avoid mistakes in the manuscript that will be published.**

Response:

The figures have now been uploaded in the appropriate order in the PDF file of the revised manuscript.
