# Supplementary material for: Higher operating theatre temperature during burn surgery increases physiological heat strain, subjective workload, and fatigue of surgical staff
Source: PLoS One. 2023 Jun 2;18(6):e0286746. doi: 10.1371/journal.pone.0286746 (PMC10237492; doi:10.1371/journal.pone.0286746)
Supplement: Attachment — Submitted filename: Reviewer comments.docx [file pone.0286746.s012.docx]

Reviewer comments

The authors completed attractive work overall, with the aims and methods section being interesting. My comments are all minimal.

1. I see cognitive performance as a complex phenomenon. Thus, analyses involving three cognitive domains provide limited explanations for something as complex as the regulation of cognitive performance. If you don't have a justification, consider using executive function performance and not cognitive performance.

2. Line 156. Working memory is not a component of executive function? Please be more explicit.

3. Line 99. I am not native to an English-speaking country, but if there is no specific reason, consider using CON and not CONTROL.

4. Line 59. Workload and fatigue already appear in the title. Consider defining other keywords. Perhaps this increases the chances of your article being found in a database.

5. Line 109. Considering the results of this study, I would suggest head cooling during burn surgery. Perhaps you should know about a recent work on the topic (doi: 10.1111/sms.13985).

6. Line 129. Consider using T_CORE_. I think it improves visualization.

7. Line 136. Why was water not provided ad libitum for the medical team?

8. Line 139. Just a suggestion. You could have corrected the BM pre- and post-test for the volume of water ingested (Please note the question before), the clothes you wore, and the weight of the experimental instruments.

9. Line 176. Why didn't you use a more up-to-date version of R Studio for Windows (*e.g.*, version 2022.07.2)? I think this may have reflected in the quality of the figures. Use colors and the ggplot2 package.

10. Line 182. I'm not a statistician, but I was concerned about Tukey's use of post hoc. I think some variables have a coefficient of variation (CV) ≥ 15%. Tukey's post hoc is very rigorous; consider using a less rigorous one for variables with a high CV. I consider in my post hoc choices the number of treatments and the CV of the variable. Thus, I minimize the chances of type I and II errors (DOI: 10.1177/0013164488483001; ISBN: 9788587144522).

Table. Determining the post-hoc test

| Variable instability | CV | Number  of treatments | Post hoc test |
| --- | --- | --- | --- |
| Low | ≤15% | ≤5 | Tukey, SNK or Scheffé |
| Low | ≤15% | >5 | SNK or Tukey |
| Medium | 15 a 30% | ≤4 | Student's t test |
| Medium | 15 a 30% | ≥5 | SNK or Duncan |
| High | ≥30% | ≤4 | Student's t test |
| High | ≥30% | >4 | Duncan |

11. Line 189. If you don't have a specific reason, consider using relative humidity instead of Relative Humidity.

12. Line 198. If you don't have a cause in mind, think about using Figure rather than Fig.

13. Lines 183, 207, and 228. Are all results shown expressed as mean ± SD or mean ± SEM?

14. Line 279. I am aware of the study by Casa et al. (2000); however, you could be a little more cautious in stating that individuals have significant dehydration (consider using hypohydration). Measurement of urine specific gravity may not reflect plasma osmolality, the most efficient measure to assess hydration status. Perhaps you should know and consider the article by Pereira et al. (2017) (DOI: 10.23736/S0022-4707.16.06836-5).

15. Lines 394 to 532. Important current references in the area of thermoregulation and areas similar to this study were not presented. You only presented three references from the last three years. Please consider citing these: DOI: 10.1371/journal.pone.0274584; DOI: 10.1038/s41572-021-00334-6; DOI: 10.1177/1553350620934931; DOI: 10.1308/rcsann.2020.7001.
